# Background features in the cytology of pancreatic neoplasms

**DOI:** 10.1002/deo2.105

**Published:** 2022-03-23

**Authors:** Kenichi Hirabayashi, Tsubasa Saika, Naoya Nakamura

**Affiliations:** ^1^ Department of Pathology Tokai University School of Medicine Kanagawa Japan; ^2^ Diagnostic Pathology Center Tokai University Hospital Kanagawa Japan

**Keywords:** background feature, cytology, mucin, necrosis, pancreatic neoplasm

## Abstract

Cytology is a useful method for diagnosing pancreatic neoplasms. Although endoscopic ultrasound‐guided fine‐needle aspiration has recently become the mainstream method for the diagnosis of pancreatic neoplasms, pancreatic juice and pancreatic duct brushing cytology continue to be useful diagnostic methods for the investigation of pancreatic neoplasms. Diagnoses using pancreatic cytology are primarily based on the features related to tumor cells; however, evaluation of the background features provides important information that could further aid the diagnosis. Pancreatic neoplasms show various histological types, each of which is associated with its own characteristic background features. The necrotic background, desmoplastic stroma, and presence of cancer‐associated fibroblasts are background features of pancreatic ductal adenocarcinoma, a mucinous background is associated with intraductal papillary mucinous neoplasms and mucinous cystic neoplasms, and hyaline globules are observed in solid pseudopapillary neoplasms. However, some background features are associated with more than one histological type of pancreatic neoplasm, highlighting the importance to base a diagnosis on the results of a comprehensive analysis of not only the background features but also the tumor cells. Here, we provide a review of the key background cytological features of pancreatic neoplasms, which can serve as a guide to improve diagnosis and research.

## INTRODUCTION

Pancreatic cancer is a malignant neoplasm with an extremely poor prognosis; thus, early diagnosis and treatment are desirable. Although the accuracy of pancreatic cancer diagnosis has improved in recent years due to the development of diagnostic imaging, it is still necessary to carry out biopsy and cytology to differentiate between benign and malignant neoplasms and to determine the histological type. Pancreatic juice cytology and pancreatic duct brushing cytology have long been the major diagnostic methods for pancreatic neoplasms; however, the recent development and application of endoscopic ultrasound‐guided fine‐needle aspiration (EUS‐FNA) has substantially aided and improved the cytological diagnosis of pancreatic neoplasms. Pancreatic juice cytology and pancreatic duct brushing cytology are mainly used for investigating neoplasms involving the large pancreatic ducts, such as pancreatic ductal adenocarcinoma (PDAC) and intraductal papillary mucinous neoplasms (IPMNs), whereas EUS‐FNA is now more commonly used for pancreatic neoplasms that have less association with the pancreatic ducts, such as neuroendocrine neoplasms (NENs), acinar cell carcinoma (ACC), serous cystic neoplasms (SCNs), mucinous cystic neoplasms (MCNs), and solid pseudopapillary neoplasms (SPNs). In cytology, the diagnosis of pancreatic neoplasms is based mainly on morphological evaluation of the tumor cells; however, background features such as the presence of necrosis or mucin also provide important information to assist with the diagnosis. In this review, we discuss the background cytological features of various histological subtypes of pancreatic neoplasms.

## NECROTIC BACKGROUND

**FIGURE 1:**
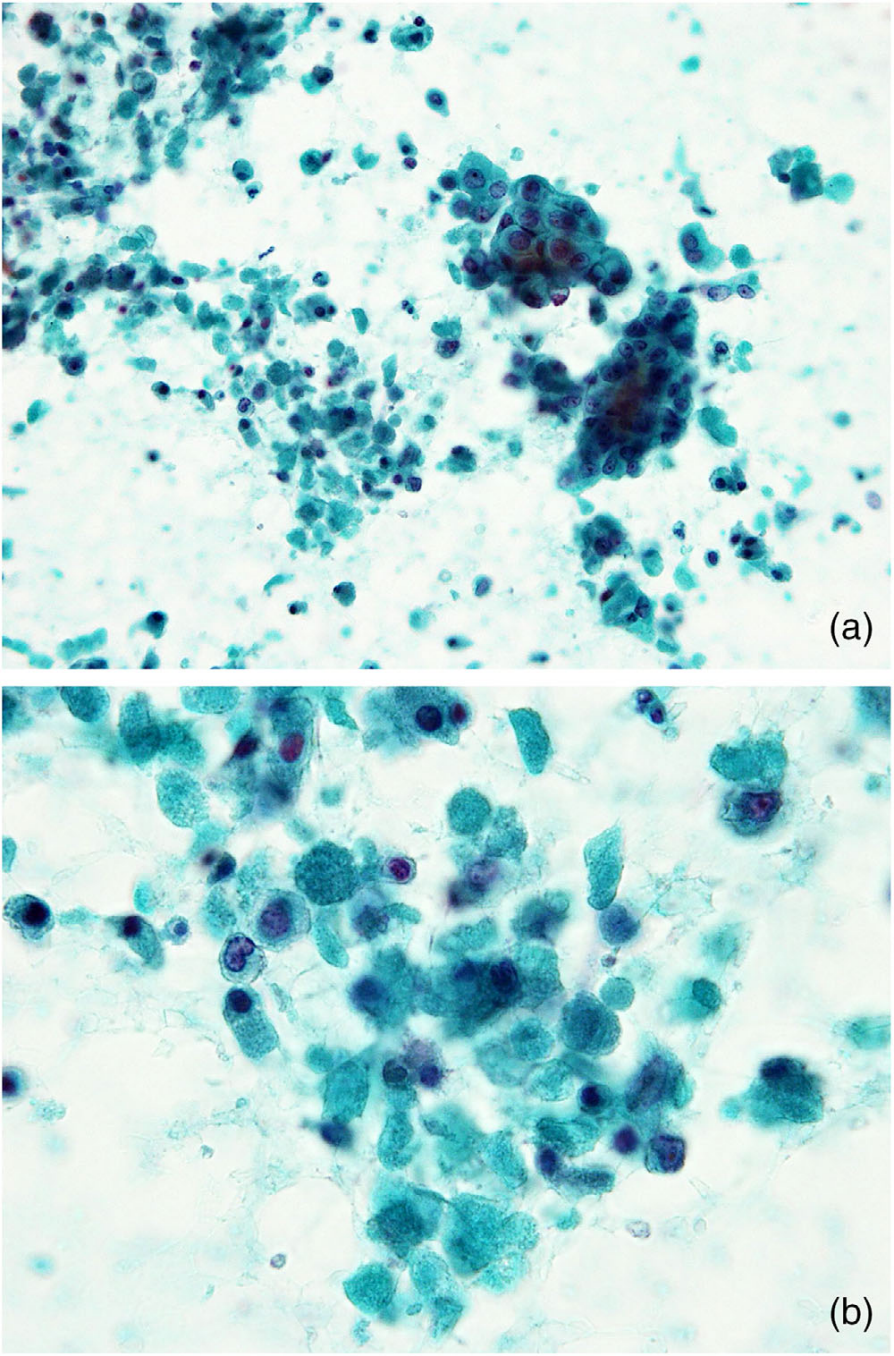
Necrosis in ductal adenocarcinoma (Papanicolaou stain). Light green‐stained necrotic material is observed in the background with tumor cells. (a) Low‐power view, (b) high‐power view

A necrotic background is one of the most common background features on the cytology of malignant tissues. Tumor necrosis is caused by ischemia or apoptosis induced by high cellular proliferation.^1^ Some authors have proposed including the necrotic background among the criteria for the diagnosis of pancreatic cancer on the cytology of FNA specimens.^2^, ^3^ Kiso et al.^3^ reported that the interobserver diagnostic agreement for a necrotic background in EUS‐FNA specimens was moderate and that the necrotic background represented one of the important findings for differentiating malignant from benign lesions. The incidence of a necrotic background in FNA specimens of pancreatic cancer has been reported to be 40–90% (Figure 1).^2^, ^3^, ^4^, ^5^, ^6^, ^7^, ^8^ However, in a study limited to well‐differentiated adenocarcinoma, the incidence of a necrotic background was as low as 7%, suggesting its low diagnostic significance.^9^ Mitsuhashi et al.^7^ reported two patterns of necrotic background appearance in cytological specimens of PDAC: (i) necrosis that appeared diffusely with various numbers of conspicuous malignant cells and (ii) necrosis that appeared focally in association with a few single cells that were either slightly atypical or evidently malignant. Mallik et al.^8^ reported that the majority of the necrosis observed was localized, occurring in less than 10% of the smear area.

The cytological background of ACC is often clean, and necrosis was only detected in 2 out of 16 cases (13%) in three studies that reported more than four cases of ACC.^10^, ^11^, ^12^, ^13^

A necrotic background is one of the findings that supports the diagnosis of a high‐grade IPMN (carcinoma *in situ*) and an IPMN with invasive carcinoma. Michaels et al.^14^ reported that a necrotic background was not observed in low‐grade IPMNs but was observed in 43% of high‐grade IPMNs and in 100% of IPMNs with invasive carcinoma, suggesting that the presence of a necrotic background is a useful finding for the diagnosis of malignant neoplasms. In a study of the cystic contents of IPMNs, a necrotic background was observed in 4% of low‐grade IPMNs and 81% of high‐grade IPMNs, and a necrotic background was the most common finding for diagnosing high‐grade IPMNs, along with an abnormal chromatin pattern and an increased nuclear‐cytoplasmic ratio (sensitivity: 80%, specificity: 96%, accuracy: 88%).^15^ Necrosis was observed in 60% (3/5 cases) of intraductal oncocytic papillary neoplasms (IOPNs), and single‐cell necrosis and areas of confluent necrosis were reported in a case of IOPN with invasive carcinoma.^16^

NENs are classified into well‐differentiated neuroendocrine tumors (NETs) and poorly differentiated neuroendocrine carcinomas (NECs); NETs are further subclassified into NET G1 (Ki67 index < 3% and/or mitotic count < 2/2 mm^2^), NET G2 (Ki67 index: 3–20% and/or mitotic count: 2–20/2 mm^2^), and NET G3 (Ki67 index >20% and/or mitotic count >20/2 mm^2^).^17^ A necrotic background is rare in NETs. Siegel et al.^18^ reported that necrosis was not observed in NET G2 and was observed in 13% of patients with NET G3. In contrast, necrosis was observed with approximately 40% of the NECs and was the most consistent cytological feature for the diagnosis of NEC.^18^, ^19^ In addition, in a study investigating the cytological features of metastatic NEN, the presence of necrosis was significantly associated with survival in the stage‐adjusted analysis.^20^

SPNs are low‐grade neoplasms, although hemorrhagic necrosis is frequently observed.^21^ Extensive necrosis has been reported to be associated with a poor prognosis.^22^ Necrosis is also frequently observed in cytological specimens, and in a review of 43 cases, necrosis was observed in 37% of the cases.^23^ Therefore, it is necessary to confirm the pseudopapillary pattern of tumor cells around the vascular cores—which are a characteristic cytological feature of SPNs—to distinguish SPNs from high‐grade neoplasms such as PDAC.

Necrosis can also be observed in nonneoplastic conditions such as pancreatitis and the formation of pancreatic pseudocysts.^24^, ^25^, ^26^ Thus, the observation of necrosis alone cannot help to distinguish between benign and malignant conditions, and it is important to confirm the diagnosis by investigating the presence of atypical cells or tumor cells. If a large number of atypical cells are found in the necrotic background, then a diagnosis of malignant neoplasms can be easily made. However, if only a few atypical cells are present, false‐negative results are more likely. Aceero et al.^27^ also reported that the presence of necrosis in the cytological specimens of pancreatic solid lesions significantly and negatively influenced the accuracy of cytological diagnoses (57% false‐negative rate). Thus, when a necrotic background is observed, investigating the presence and frequency of atypical cells and tumor cells is essential for an accurate diagnosis.

## MUCINOUS BACKGROUND

**FIGURE 2:**
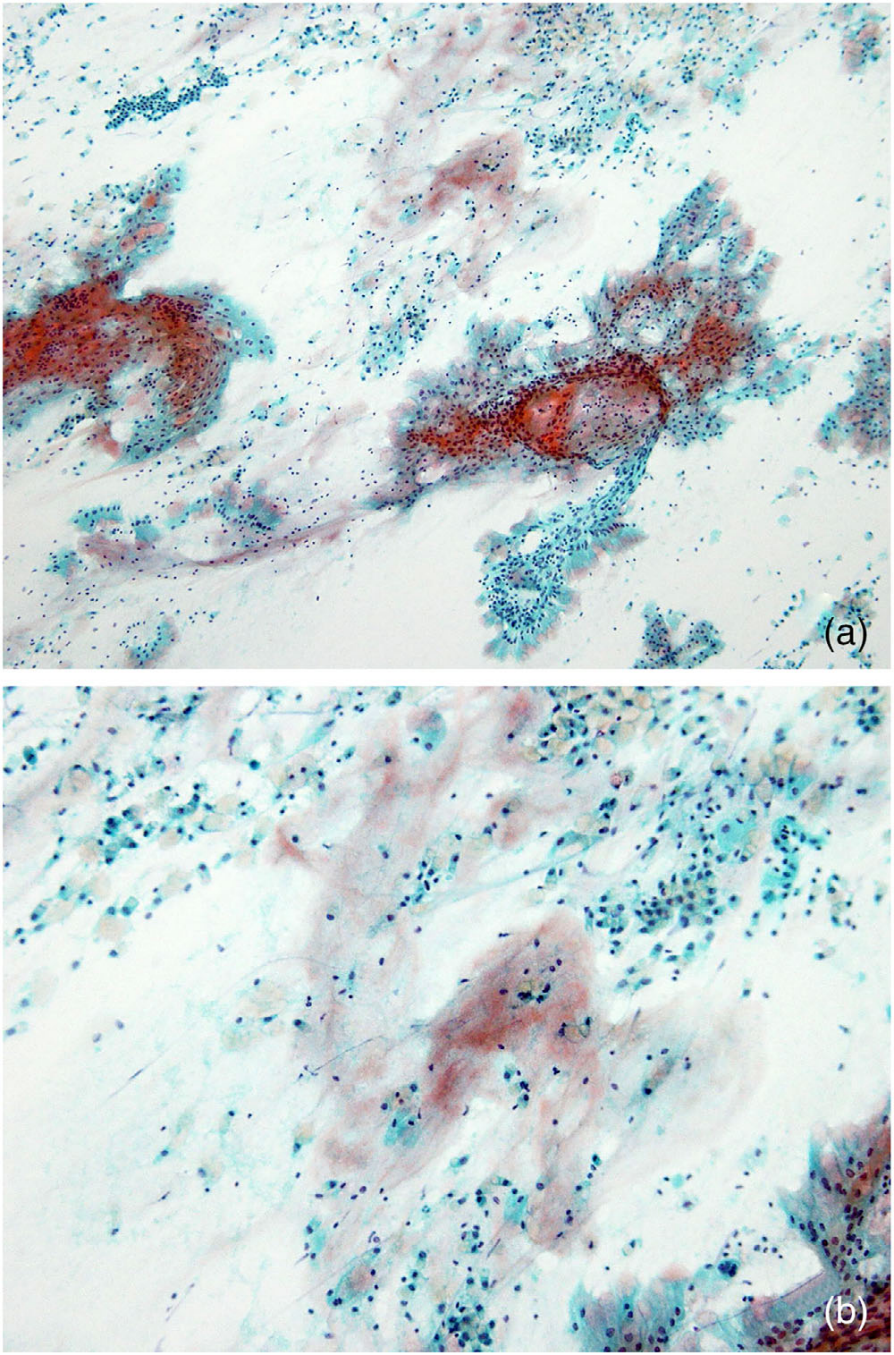
Mucinous background in intraductal papillary mucinous neoplasm (Papanicolaou stain). A large amount of thick, eosin‐stained mucus appears in the background of papillary tumor cell clusters. (a) Low‐power view, (b) high‐power view

A mucinous background is one of the diagnostic findings of mucin‐producing neoplasms such as IPMNs, MCNs, and colloid carcinomas and is useful in differentiating from nonmucin‐producing neoplasms such as SCN, SPN, NEN, and ACC (Figure 2).^28^, ^29^ Molecular analysis of the contents of cystic neoplasms has also been carried out, and mutations in *KRAS, GNAS*, and *RNF43* have been found to serve as markers of mucinous neoplasms, especially *GNAS* mutations that serve as diagnostic markers of IPMNs.^30^, ^31^, ^32^, ^33^, ^34^, ^35^, ^36^ However, FNA of pancreatic cystic neoplasms is rarely performed in Japan because of concerns regarding the peritoneal dissemination of tumor cells.

Kiso et al.^3^ investigated the EUS‐FNA specimens of 111 PDAC and 31 benign cases, including seven low‐grade to intermediate‐grade IPMNs. A mucinous background was observed in the tissue samples of 61% of the PDAC cases and 74% of the benign cases, with no significant difference between them. Mallik et al.^8^ reported that a mucinous background was observed in 9% of the cases assessed on a review of 89 EUS‐FNA specimens of PDAC. If abundant mucus is observed in the background of FNA specimens of solid neoplasms, colloid carcinoma should be considered.

IPMN and MCN show a rich mucinous background, whereas the background of intraductal tubulopapillary neoplasm and IOPN shows comparatively less mucin than that of IPMN and MCN.^16^, ^28^, ^37^ On the analysis of IOPN, extracellular mucin was observed in the smear background in only 40% (2/5) of the cases.^16^

No significant difference was found in the frequency of observing a thick mucinous background in IPMNs according to atypia or invasion (low‐grade IPMN: 53%, high‐grade IPMN: 57%, IPMN with invasive carcinoma: 50%).^14^

EUS‐FNA specimens obtained by transgastric or transduodenal puncture may contain gastric or duodenal mucus.^4^, ^8^, ^29^ Therefore, care should be taken not to misinterpret the contamination of gastric duodenal mucus as the mucinous background of pancreatic neoplasms when assessing the findings of EUS‐FNA cytology. In general, pancreatic neoplasms have thick, colloidal mucin, whereas gastroduodenal mucin is more watery and colloid‐like.^38^ However, it is difficult to distinguish gastroduodenal mucin from pancreatic neoplasm mucin; therefore, it is important to carefully evaluate cellular morphology and other factors for diagnosis. In addition, the mucinous background is easier to evaluate with conventional smears than with liquid‐based cytology.^38^

### Desmoplastic stroma, cancer‐associated fibroblasts, and stromal fragments

Abundant fibrous and desmoplastic stroma are characteristic histological features of PDAC. However, it has been reported that the presence of desmoplastic stroma in cytological specimens is associated with false‐negative results in the diagnosis of pancreatic cancer because desmoplastic stroma makes it more difficult to obtain representative tissue samples for analysis and can be confused with chronic pancreatitis (46% false‐negative rate).^27^ Desmoplastic stroma is composed of myofibroblasts, collagen fibers, proteoglycans, and glycoproteins.^39^, ^40^ Cancer‐associated fibroblasts (CAFs), one of the components of desmoplastic stroma, are activated fibroblasts and myofibroblasts found in the periphery of cancer cells and play an important role in invasion and metastasis.^41^, ^42^, ^43^ Morphologically, CAFs are spindle‐shaped cells with a distinct nucleolus and rich cytoplasm; CAFs express alpha‐smooth muscle actin, and the expression of nectin‐1 and podoplanin in CAFs has been reported to be associated with poor prognosis in PDAC patients.^42^, ^43^

**FIGURE 3:**
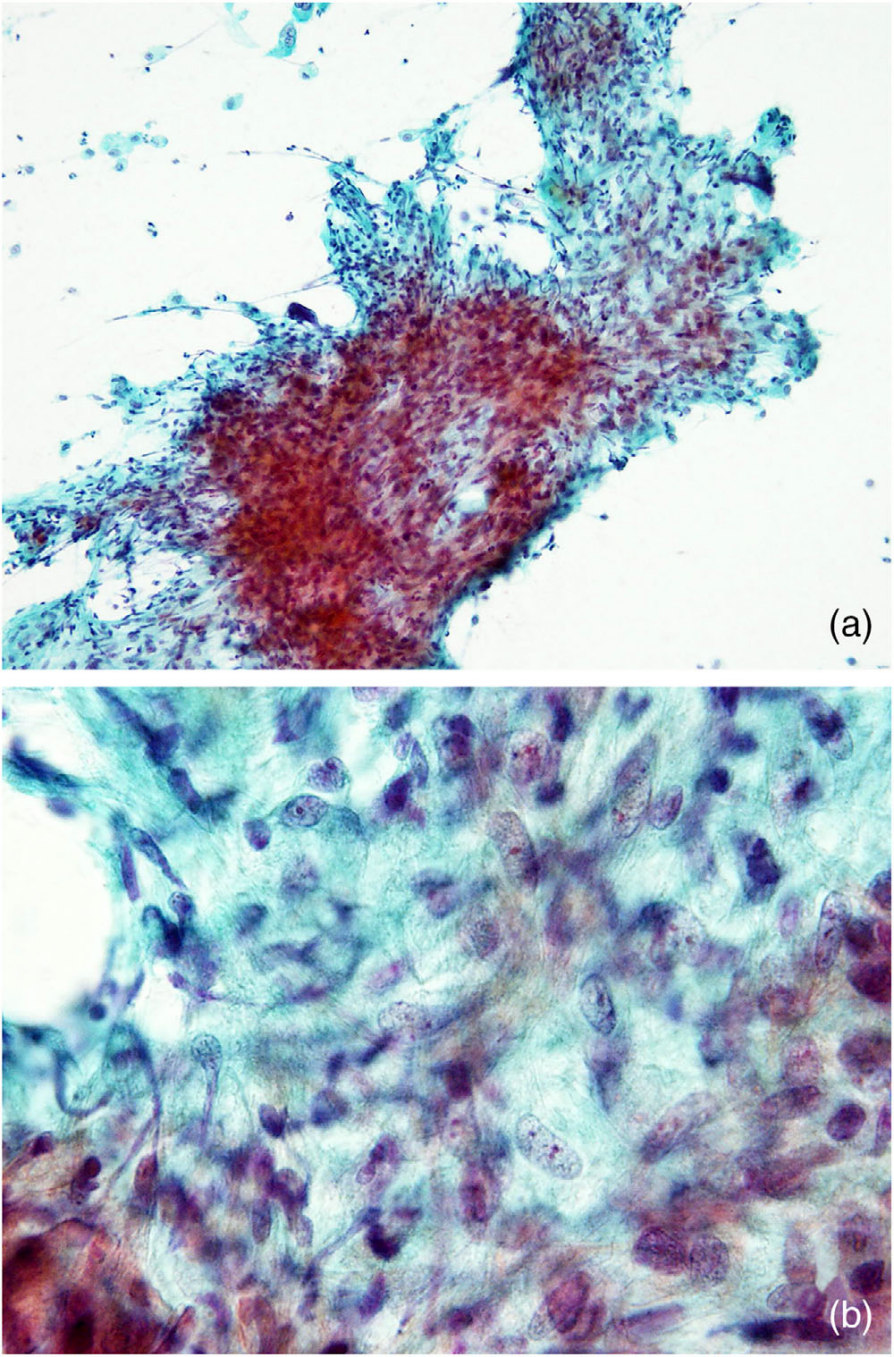
Cancer‐associated fibroblasts (CAFs) in ductal adenocarcinoma (Papanicolaou stain). Spindle‐shaped cells with large oval nuclei and prominent nucleoli showing storiform proliferation. (a) Low‐power view, (b) high‐power view

　Although desmoplastic stroma and CAFs are also observed in cytological specimens of PDACs, they have not been studied in detail. We previously investigated the diagnostic significance of CAFs in pancreatic duct brushing cytology.^44^ In this study, we compared the prevalence and nuclear size of the fibrous stroma, including CAFs, in PDACs and benign cases. Fibrous stroma was found in 60% of PDACs and in 24% of benign cases, and the mean nuclear size of the fibrous stroma in PDAC cases was significantly larger than that in benign cases (cut off: 10.22 μm). In addition, the fibrous stroma in PDAC cases with nuclei larger than 10.22 μm had clearly prominent nucleoli. In contrast, the fibrous stroma in benign cases did not have clear nucleoli. Based on these results, we defined CAFs in cytological specimens as spindle‐shaped cells with nuclei larger than 10.22 μm and prominent nucleoli (Figure 3). In addition, the presence of CAFs in pancreatic duct brushing cytological specimens showed 100% positive predictive value and specificity in the diagnosis of PDAC, indicating that CAFs may be useful in the diagnosis of PDAC.

Stromal fragments also appear in benign diseases such as SCN, autoimmune pancreatitis (AIP), and chronic pancreatitis.^45^, ^46^ Deshpande et al.^45^ reported that stromal fragments were observed in 88% (14/16) of AIP cases, 37% (7/19) of chronic pancreatitis not otherwise specified (NOS) cases, and 75% (12/16) of PDAC cases. Furthermore, they reported that the incidence of stromal fragments with embedded lymphocytes (greater than 30 per 60 × field) was significantly higher in AIP than in PDAC and chronic pancreatitis (AIP: 38%; PDAC: 13%; pancreatitis, NOS: 0%). These findings, together with clinical and radiological findings, could be diagnostic of AIP.^45^

### Hyaline globules

**FIGURE 4:**
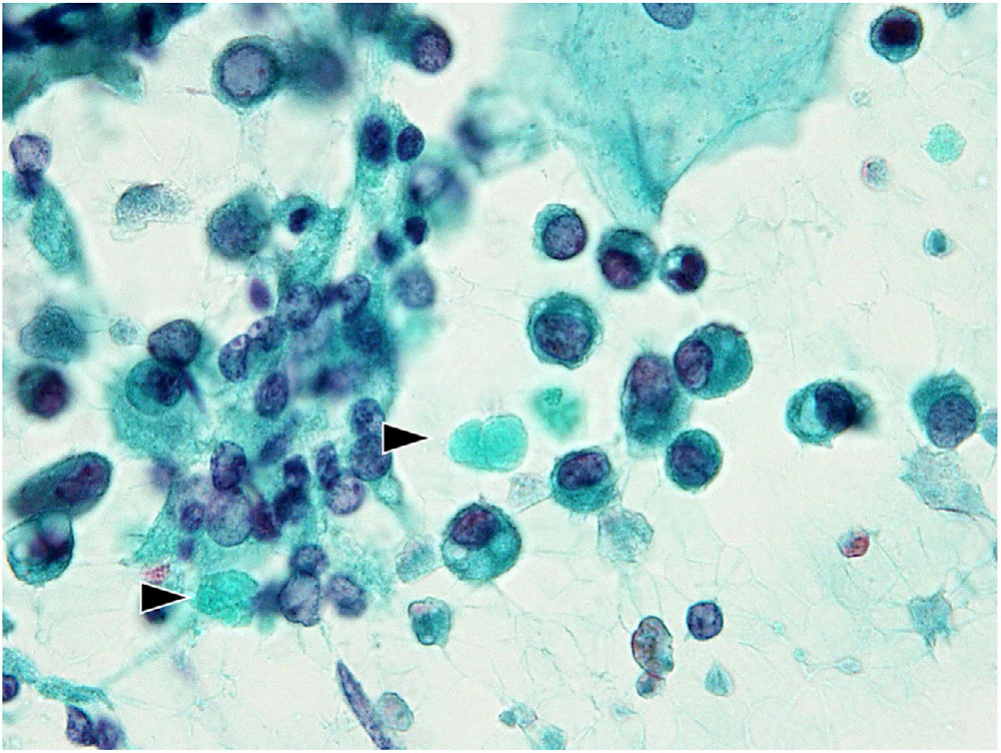
Hyalin globules in solid‐pseudopapillary neoplasm (Papanicolaou stain). Unstructured, globular materials well‐dyed as light green evident in the background (arrowhead)

Hyaline globules are microspheres that stain eosinophilic on hematoxylin‐eosin staining and light green on Papanicolaou staining and are located in either the intracytoplasmic or extracytoplasmic regions in tumor cells (Figure 4). Hyaline globules are Periodic acid Schiff‐positive/diastase‐resistant and positive for alpha‐1‐antitrypsin.^47^ In EUS‐FNA studies, hyaline globules were observed in 39% of SPNs but not in NETs, which is a useful finding for differentiating SPNs from NETs.^48^ However, in a study of histological specimens, globules were observed in 5% of NETs.^47^ Hyaline globules have also been reported in histological specimens of pancreatic metastases of hepatocellular carcinoma, hepatoid pancreatic carcinoma, and IOPN.^49^, ^50^, ^51^

### Calcification

Calcification is often observed in pancreatic neoplasms, especially in SPNs and SCNs. The incidence of calcification in cytological specimens has been reported to be 39% (7/18) in SPN cases, 14% (4/28) in SCN cases, 5% (1/20) in NET cases, and 0% (0/4) in ACC cases.^13^, ^46^ Psammomatous calcifications have been reported in the smear background of a case of IOPN.^16^

### Other features

In SPNs, along with necrosis and calcification, various degenerative background features, such as foam cell clusters, cholesterol clefts, amorphous granular background, and histiocytic giant cells, are obtained.^48^ A background magenta matrix and degenerative background are more frequent in SPN than in NETs and are thus useful in differentiating between them (magenta matrix: SPN = 89%, NET = 0%; degenerative background: SPN = 89%, NET = 20%).^48^ A case of NET with fibrous extracellular spheroids and three NET cases with abundant amyloid in the background have been reported.^52^, ^53^ Cholesterol crystals can be observed in SPNs and lymphoepithelial cysts.^13^, ^54^, ^55^

## CONCLUSION

There are various histological types of pancreatic neoplasms, and characteristic background features for each type that may be useful for diagnosis have been identified. However, as certain background features also overlap between histological types, it is important to make a comprehensive diagnosis by carefully observing not only the background features but also the characteristics of tumor cells and atypical cells. The diagnostic utility of background features, such as necrotic background or CAFs, in pancreatic juice cytology and/or pancreatic duct brushing cytology in early‐stage PDAC with pancreatic duct stenosis or dilation is still not well understood. Therefore, further studies on the diagnostic utility of background findings in early‐stage PDAC are needed.

## CONFLICT OF INTEREST

KH is an Associate Editor of DEN Open.

## FUNDING

None
